# Supplementary material for: COZOID: contact zone identifier for visual analysis of protein-protein interactions
Source: BMC Bioinformatics. 2018 Apr 6;19:125. doi: 10.1186/s12859-018-2113-6 (PMC5889581; doi:10.1186/s12859-018-2113-6)
Supplement: Supplementary file 2 — Software build. Executable binary file of the software tool. (ZIP 118,784 kb) [file 12859_2018_2113_MOESM2_ESM.zip › cozoid/java/docs/beansbinding-1.2.1-doc.zip!/org/jdesktop/el/ELContext.html]

ELContext (Beans Binding)


---


|  |  |  |
| --- | --- | --- |
| **Overview** \| **Package** \| **Class** \| **Use** \| **Tree** \| **Deprecated** \| **Index** \| **Help** | |  |
| **PREV CLASS**   **NEXT CLASS** | **FRAMES**    **NO FRAMES**     **All Classes** |  |
| SUMMARY: NESTED \| FIELD \| CONSTR \| METHOD | DETAIL: FIELD \| CONSTR \| METHOD |  |


---


## org.jdesktop.el Class ELContext

```
java.lang.Object
  org.jdesktop.el.ELContext
```

**Direct Known Subclasses:**: EvaluationContext

---

``` public abstract class ELContext extends java.lang.Object ```

Context information for expression evaluation.

To evaluate an `Expression`, an `ELContext` must be
provided. The `ELContext` holds:

- a reference to the base `ELResolver` that will be consulted
  to resolve model objects and their properties
- a reference to `FunctionMapper` that will be used
  to resolve EL Functions.- a reference to `VariableMapper` that will be used
    to resolve EL Variables.- a collection of all the relevant context objects for use by
      `ELResolver`s
    - state information during the evaluation of an expression, such as
      whether a property has been resolved yet

The collection of context objects is necessary because each
`ELResolver` may need access to a different context object.
For example, JSP and Faces resolvers need access to a
`javax.servlet.jsp.JspContext` and a
`javax.faces.context.FacesContext`, respectively.

Creation of `ELContext` objects is controlled through
the underlying technology. For example, in JSP the
`JspContext.getELContext()` factory method is used.
Some technologies provide the ability to add an `ELContextListener`
so that applications and frameworks can ensure their own context objects
are attached to any newly created `ELContext`.

Because it stores state during expression evaluation, an
`ELContext` object is not thread-safe. Care should be taken
to never share an `ELContext` instance between two or more
threads.

**Since:**
:   JSP 2.1

**See Also:**: `ELContextListener`, `ELContextEvent`, `ELResolver`, `FunctionMapper`, `VariableMapper`, `javax.servlet.jsp.JspContext`

---

| **Field Summary** | |
| --- | --- |
| `static java.lang.Object` | `UNRESOLVABLE_RESULT` |


**Constructor Summary**

`ELContext()`


| **Method Summary** | |
| --- | --- |
| `java.lang.Object` | `getContext(java.lang.Class key)`             Returns the context object associated with the given key. |
| `abstract  ELResolver` | `getELResolver()`             Retrieves the `ELResolver` associated with this context. |
| `abstract  FunctionMapper` | `getFunctionMapper()`             Retrieves the `FunctionMapper` associated with this `ELContext`. |
| `java.util.Locale` | `getLocale()`             Get the `Locale` stored by a previous invocation to `setLocale(java.util.Locale)`. |
| `abstract  VariableMapper` | `getVariableMapper()`             Retrieves the `VariableMapper` associated with this `ELContext`. |
| `boolean` | `isPropertyResolved()`             Returns whether an `ELResolver` has successfully resolved a given (base, property) pair. |
| `void` | `putContext(java.lang.Class key, java.lang.Object contextObject)`             Associates a context object with this `ELContext`. |
| `void` | `setLocale(java.util.Locale locale)`             Set the `Locale` for this instance. |
| `void` | `setPropertyResolved(boolean resolved)`             Called to indicate that a `ELResolver` has successfully resolved a given (base, property) pair. |

**Methods inherited from class java.lang.Object**

`clone, equals, finalize, getClass, hashCode, notify, notifyAll, toString, wait, wait, wait`

**Field Detail**

### UNRESOLVABLE\_RESULT

```
public static final java.lang.Object UNRESOLVABLE_RESULT
```


**Constructor Detail**

### ELContext

```
public ELContext()
```


**Method Detail**

### setPropertyResolved

```
public void setPropertyResolved(boolean resolved)
```

:   Called to indicate that a `ELResolver` has successfully
    resolved a given (base, property) pair.

    The `CompositeELResolver` checks this property to determine
    whether it should consider or skip other component resolvers.

    :   **Parameters:**: `resolved` - true if the property has been resolved, or false if not. **See Also:**: `CompositeELResolver`

---


### isPropertyResolved

```
public boolean isPropertyResolved()
```

:   Returns whether an `ELResolver` has successfully resolved a
    given (base, property) pair.

    The `CompositeELResolver` checks this property to determine
    whether it should consider or skip other component resolvers.

    :   **Returns:**: true if the property has been resolved, or false if not. **See Also:**: `CompositeELResolver`

---


### putContext

```
public void putContext(java.lang.Class key,
                       java.lang.Object contextObject)
```

:   Associates a context object with this `ELContext`.

    The `ELContext` maintains a collection of context objects
    relevant to the evaluation of an expression. These context objects
    are used by `ELResolver`s. This method is used to
    add a context object to that collection.

    By convention, the `contextObject` will be of the
    type specified by the `key`. However, this is not
    required and the key is used strictly as a unique identifier.

    :   **Parameters:**: `key` - The key used by an @{link ELResolver} to identify this context object.: `contextObject` - The context object to add to the collection. **Throws:**: `java.lang.NullPointerException` - if key is null or contextObject is null.

---


### getContext

```
public java.lang.Object getContext(java.lang.Class key)
```

:   Returns the context object associated with the given key.

    The `ELContext` maintains a collection of context objects
    relevant to the evaluation of an expression. These context objects
    are used by `ELResolver`s. This method is used to
    retrieve the context with the given key from the collection.

    By convention, the object returned will be of the type specified by
    the `key`. However, this is not required and the key is
    used strictly as a unique identifier.

    :   **Parameters:**: `key` - The unique identifier that was used to associate the context object with this `ELContext`. **Returns:**: The context object associated with the given key, or null if no such context was found. **Throws:**: `java.lang.NullPointerException` - if key is null.

---


### getELResolver

```
public abstract ELResolver getELResolver()
```

:   Retrieves the `ELResolver` associated with this context.

    The `ELContext` maintains a reference to the
    `ELResolver` that will be consulted to resolve variables
    and properties during an expression evaluation. This method
    retrieves the reference to the resolver.

    Once an `ELContext` is constructed, the reference to the
    `ELResolver` associated with the context cannot be changed.

    :   **Returns:**: The resolver to be consulted for variable and property resolution during expression evaluation.

---


### getFunctionMapper

```
public abstract FunctionMapper getFunctionMapper()
```

:   Retrieves the `FunctionMapper` associated with this
    `ELContext`.

    :   **Returns:**: The function mapper to be consulted for the resolution of EL functions.

---


### getLocale

```
public java.util.Locale getLocale()
```

:   Get the `Locale` stored by a previous invocation to
    `setLocale(java.util.Locale)`. If this method returns non `null`,
    this `Locale` must be used for all localization needs
    in the implementation. The `Locale` must not be cached
    to allow for applications that change `Locale` dynamically.

    :   **Returns:**: The `Locale` in which this instance is operating. Used primarily for message localization.

---


### setLocale

```
public void setLocale(java.util.Locale locale)
```

:   Set the `Locale` for this instance. This method may be
    called by the party creating the instance, such as JavaServer
    Faces or JSP, to enable the EL implementation to provide localized
    messages to the user. If no `Locale` is set, the implementation
    must use the locale returned by `Locale.getDefault( )`.

---


### getVariableMapper

```
public abstract VariableMapper getVariableMapper()
```

:   Retrieves the `VariableMapper` associated with this
    `ELContext`.

    :   **Returns:**: The variable mapper to be consulted for the resolution of EL variables.


---


|  |  |  |
| --- | --- | --- |
| **Overview** \| **Package** \| **Class** \| **Use** \| **Tree** \| **Deprecated** \| **Index** \| **Help** | |  |
| **PREV CLASS**   **NEXT CLASS** | **FRAMES**    **NO FRAMES**     **All Classes** |  |
| SUMMARY: NESTED \| FIELD \| CONSTR \| METHOD | DETAIL: FIELD \| CONSTR \| METHOD |  |


---
